# Lobeglitazone Exerts Anti-Inflammatory Effect in Lipopolysaccharide-Induced Bone-Marrow Derived Macrophages

**DOI:** 10.3390/biomedicines9101432

**Published:** 2021-10-10

**Authors:** Dabin Jeong, Wan-Kyu Ko, Seong-Jun Kim, Gong-Ho Han, Daye Lee, Seung-Hun Sheen, Seil Sohn

**Affiliations:** 1 Department of Neurosurgery, CHA Bundang Medical Center, Seongnam-si 13496, Korea; dbjekqls77@gmail.com (D.J.); wankyu@chauniv.ac.kr (W.-K.K.); ksj987456@chauniv.ac.kr (S.-J.K.); hgh429@chauniv.ac.kr (G.-H.H.); day03@chauniv.ac.kr (D.L.); 2 Department of Biology, Lawrence University, Appleton, WI 54911, USA; 3 Department of Biomedical Science, CHA University, Seongnam-si 13493, Korea

**Keywords:** bone-marrow derived macrophages, lipopolysaccharide, lobeglitazone, anti-inflammation, mitogen-activated protein kinase

## Abstract

The purpose of this study is to elucidate the anti-inflammatory effect of lobeglitazone (LOBE) in lipopolysaccharide (LPS)-induced bone-marrow derived macrophages (BMDMs). We induced nitric oxide (NO) production and pro-inflammatory gene expression through LPS treatment in BMDMs. The changes of NO release and expression of pro-inflammatory mediators by LOBE were assessed via NO quantification assay and a real-time quantitative polymerase chain reaction (RT-qPCR), respectively. In addition, the regulatory effect of LOBE on activation of mitogen-activated protein kinase (MAPK) signaling pathway was investigated by measuring the phosphorylation state of extracellular regulatory protein (ERK) and c-Jun N-terminal kinase (JNK) proteins by Western blot. Our results show that LOBE significantly reduced LPS-induced NO production and pro-inflammatory gene expression of interleukin-1β (IL-1β), IL-6, inducible nitric oxide synthase (iNOS), cyclooxygenase-2 (COX-2), and monocyte chemoattractant protein-1 (MCP-1). Moreover, LOBE reduced phosphorylation levels of ERK and JNK of MAPK signaling pathway. In conclusion, LOBE exerts an anti-inflammatory effect in LPS-induced BMDMs by suppression of NO production and pro-inflammatory gene expression, and this effect is potentially through inhibition of the MARK signaling pathway.

## 1. Introduction

Inflammation is the host’s defensive response against pathogenic infection, cellular stress, and tissue injury [1], and macrophages are critical players in modulation of inflammation. They survey pathogen invasion and respond to cellular stress by releasing a variety of pro-inflammatory mediators such as nitric oxide (NO), interleukin-1β (IL-1β), IL-6, cyclooxygenase-2 (COX-2), and monocyte chemoattractant protein-1 (MCP-1) [2,3,4,5,6,7].

Upregulation of macrophage inflammation is characterized by aberrant increase of pro-inflammatory mediators. Indeed, accumulation of aforementioned pro-inflammatory molecules is implicated in pathogenesis of autoimmune and inflammatory diseases such as type 2 diabetes, atherosclerosis, and spinal cord injury [8,9,10]. Current pharmaceutical interventions including use of corticosteroid and non-steroidal anti-inflammatory drugs (NSAIDs) are commonly practiced for managing inflammation [11,12]. However, off-target events and adverse side effects of current anti-inflammatory drug imply unmet demand for identification of a new drug with potential anti-inflammatory properties [13].

Lobeglitazone (LOBE) is a potential peroxisome-proliferator-activated receptor α/γ (PPARα/γ) agonist and a newly developed synthetic thiazolidinedione (TZD) class drug for restoration of insulin sensitivity in type 2 diabetes patients [14]. Although primary function of TZDs is anti-diabetic, TZDs are also reported to possess anti-inflammatory effects [15]. LOBE, an emerging TZD, is extensively studied for its anti-diabetic effects in both pre-clinical and clinical trials [16,17,18,19]. However, its anti-inflammatory assessment was limited to adipose tissue and human umbilical vein endothelial cells (HUVECs), leaving regulatory effect of LOBE on macrophage inflammation not elucidated [18,20].

The purpose of the present study is to examine anti-inflammatory action of LOBE in macrophage inflammation. We employed rat bone-marrow derived macrophages (BMDMs). Lipopolysaccharide (LPS) was used as a stimulant to induce inflammation in macrophages in vitro [21,22]. In this study, the effect of LOBE on expression of pro-inflammatory mediators and mitogen-activated protein kinase (MAPK) pathway were evaluated. Our results demonstrate that LOBE down-regulated key pro-inflammatory mediators at transcription level, potentially through inhibition of extracellular regulatory protein (ERK) and c-Jun N-terminal kinase (JNK) signaling of MAPK pathway. These findings can provide insight into the pharmacological role of LOBE in macrophage inflammation, and its potential applications in a variety of inflammatory diseases.

## 2. Materials and Methods

### 2.1. Preparation of LOBE and LPS

The LOBE sulfate was obtained from Chong Kun Dang pharmaceutical corp. (Seoul, Korea). It was dissolved in dimethyl sulfoxide (DMSO, Thermo Scientific, Waltham, MA, USA) and was stored at room temperature in the absence of light. For in vitro assays, LOBE sulfate was solubilized in Dulbecco’s Modified Eagle’s Medium (DMEM, Gibco, Waltham, MA, USA) containing 10% fetal bovine serum (FBS, Gibco) and 1% penicillin-streptomycin (PS, Gibco). LPS was purchased from Sigma-Aldrich (Sigma, St. Louis, MI, USA) and diluted with distilled water (100 µg/mL). For subsequent experiments, it was diluted with DMEM media to a final concentration of 0.1 µg/mL LPS to induce an inflammatory response in BMDMs.

### 2.2. Experimental Group

The differences in six groups were compared: a group without any treatment in BMDMs (control group), a group only with LOBE treatment (200 µM LOBE group), a group only with LPS treatment (LPS group), and a group with LPS treatment together with indicated concentrations of LOBE (LPS + LOBE 50 µM, LPS + LOBE 100 µM, LPS + LOBE 200 µM group).

### 2.3. Cell Culture

BMDMs were cultured in DMEM containing 10% FBS, 1% PS, and 10 ng/mL of macrophage colony-stimulating factor (M-CSF) (Peprotech, Rocky Hill, NJ, USA) at 37 °C in a 5% ${\mathrm{CO}}_{2}$ atmosphere, and the culture media was changed once in 2 days.

### 2.4. BMDM Preparation

The preparation procedures of BMDMs were followed as described elsewhere [21,23,24]. First, bone-marrow monocyte (BMM) progenitor cells were extracted from pairs of tibia and femur of Sprague Dawley rats at 4 weeks of age. The inner cavities of the tibia and femur were rinsed with DMEM media supplemented with 10% FBS and 1% PS. The flushed media was passed through 70 µm cell filter. The cell suspension containing BMMs was centrifuged, and the resulting cell pellet was subjected to Red Blood Cell lysis buffer (Sigma) for 30 s. The pellet was resuspended with fresh media and cultivated for 4 h to selectively collect suspending BMMs from adherent population of cells. The BMMs were then stimulated with M-CSF (10 ng/mL) to yield BMDMs. On day 3, additional 10 mL of DMEM media supplemented with 30 ng/mL of M-CSF was added to enhance BMDM differentiation, and the cells were cultured for another four days. On day 7, the differentiated BMDMs were used in further experiments.

### 2.5. Cell Cytotoxicity Test

The procedures were followed as described previously [21]. The cell viability in various concentrations of LOBE (0, 1, 5, 10, 50, 100, 200, and 500 µM) was evaluated using a cell viability kit (EZ-Cytox, Daeil Labservice, Seoul, Korea). BMDM cells were seeded on 96 well culture plate (Falcon Becton Dickinson, Lincoln Park, NJ, USA, 4 × $10^{4}$ cells/well, *n* = 3 per group) and treated with indicated concentrations of LOBE for 24 h. At 24 h, 10-fold diluted EZ-Cytox solution was added to react for 1 h. The supernatant was assessed at 450 nm using a microplate reader (Bio-Rad, Hercules, CA, USA). The cell viability in LOBE groups was calculated as relative % normalized to control group by setting control group as 100% viability.

### 2.6. NO Quantification

NO quantification was performed as described in our previous paper [21]. BMDMs were seeded on 6 well culture plate (Falcon, 5 × $10^{5}$ cells/well, *n* = 3 per group) and treated with 50 µM, 100 µM, and 200 µM of LOBE and LPS for 24 h. At 24 h, supernatants were collected, and NO was quantified by using a Griess Reagent Kit (Thermo).

### 2.7. RT-qPCR

RT-qPCR procedures were followed as described previously [25]. BMDMs were seeded on 6 well culture plate (Falcon, 5 × $10^{5}$ cells/well, *n* = 3 per group) and treated with or without LOBE and LPS stimulation for 4 h. The total RNA of BMDMs was isolated, and a complementary sequence of the extracted RNA was synthesized to make a cDNA template. Pair of primer sequences was designed to amplify target gene products (Table 1). The RT-qPCR reaction was performed using an ABI Step One Real-time PCR System (Applied Biosystems, Warrington, UK). The RT-qPCR was run at the following conditions: 40 cycles of denaturation at 95 °C for 15 s, followed by 60 °C for 30 s to allow for extension and amplification of the target sequence. The relative expression levels of target genes were normalized to glyceraldehyde 3-phosphate dehydrogenase (GAPDH) using $2^{{-\Delta \Delta C}_{T}}$ method [26].

### 2.8. Western Blot

The detailed procedures for Western blot are described in our previous work [27]. BMDMs were seeded on 6 well culture plate (Falcon, 1 × $10^{6}$ cells/well, *n* = 3 per group) and treated with indicated concentrations of LOBE and LPS for 24 h. At 24 h, the total proteins were extracted using ice-cold RIPA lysis buffer with protease inhibitor (Roche Applied Science, Indianapolis, IN, USA) and phosphatase inhibitor cocktails (Sigma). The concentrations of isolated proteins were quantified using BCA assay kit (Thermo). Then, 20 µg of protein samples in equal volume was subjected to 10% of sodium dodecyl sulphate-polyacrylamide (SDS-PAGE) gel electrophoresis and transferred to nitrocellulose transfer membranes (Protran, Whatman, Buckinghamshire, UK). Then, 5% of bovine serum albumin (BSA) blocking solution was used to block membranes for 1 h. Primary antibodies of phosphorylated forms of ERK (p-ERK) (1:1000) and JNK (p-JNK) (1:1000) were incubated overnight and then treated with anti-rabbit secondary antibody (1:5000, Gene Tex International Corporation, Hsinchu, Taiwan, China). The same membranes were re-probed with total forms of ERK (t-ERK) (1:1000) and JNK (t-JNK) (1:1000) after stripping antibodies. The membrane bands were developed using horseradish peroxidase (Amersham™ ECL, Buckinghamshire, UK) through a G: Box Chemi-XX6 gel doc system (Syngene, Frederick, MD, USA). All primary antibodies were purchased from Cell Signaling Technology (Danvers, MA, USA). The band intensities for phosphorylated and total forms were quantified using ImageJ by National Institute of Health (NIH, Bethesda, MD, USA). Total forms were used as an internal control to normalize phosphorylation levels. ERK and JNK phosphorylation were compared between groups by setting p/t volume in LPS group as 1-fold.

### 2.9. Statistical Analysis

All values were presented as the mean ± standard deviation (SD). A one-way analysis of variance (ANOVA) followed by post hoc test (Tukey’s test) was used to compare and verify statistical differences among the groups. Differences in *p*-values for which * *p* < 0.05, ** *p* < 0.01, and *** *p* < 0.001 were considered to be statistically significant.

## 3. Results

### 3.1. Molecular Structure of LOBE and Cytotoxicity of LOBE on BMDMs

**Figure 1:**
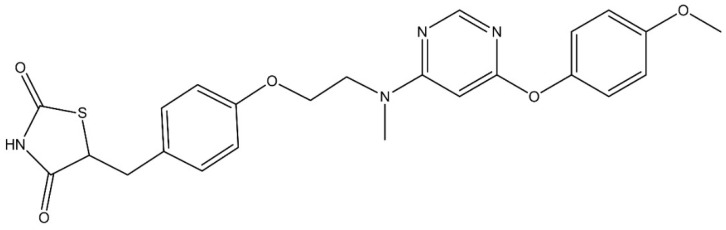
The molecular structure of lobeglitazone (LOBE).

**Figure 2:**
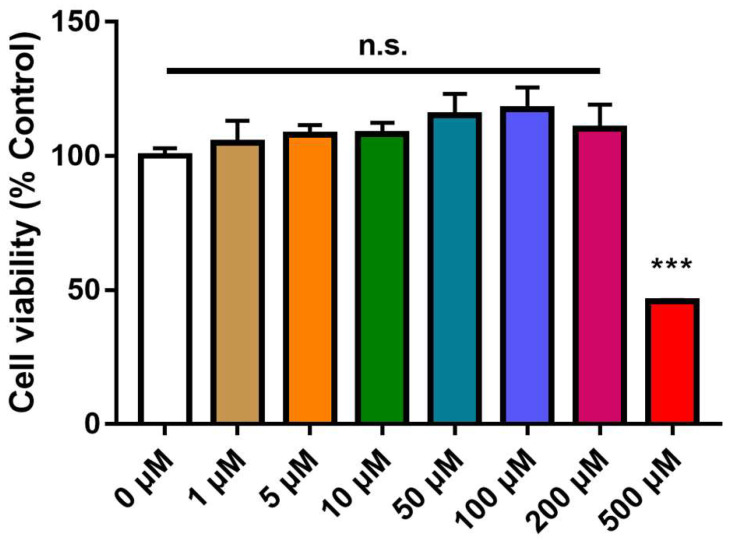
Cytotoxic effect on bone-marrow derived macrophages (BMDMs) by LOBE. BMDMs were treated with pre-determined concentrations of LOBE (0, 1, 5, 10, 50, 100, 200, and 500 µM) for 24 h. Adherent or living cells were counted using cell viability kit and normalized to control group as 100%. Results are the mean ± standard deviation (SD) of triplicate experiments: not significant (n.s.), *** *p* < 0.001, significant difference as compared to the control group and each group by a one-way analysis of variance (ANOVA) followed by Tukey’s post hoc analysis.

Figure 1 demonstrates the molecular structure of LOBE. To exclude the possibility of a drug cytotoxicity affecting investigation of the anti-inflammatory effect, the cytotoxicity of LOBE was evaluated. LOBE doses ranging from 1 µM to 200 µM did not significantly increase nor decrease cell viability (Figure 2, control: 100% ± 4.09, control vs. LOBE 1 µM: 110.35% ± 9.92, LOBE 5 µM: 108.19% ± 4.36, LOBE 10 µM: 111.35% ± 4.48, LOBE 50 µM: 118.48% ± 7.24, LOBE 100 µM: 123.4% ± 4.73, LOBE 200 µM: 115.36% ± 9.74, not significant: n.s.). However, a significant decline was observed in 500 µM of LOBE (Control vs. LOBE 500 µM: 100% ± 4.09 vs. 46.86% ± 0.62, *** *p* < 0.001). Three concentrations 50 µM, 100 µM, and 200 µM of LOBE were selected and used in subsequent experiments.

### 3.2. Inhibitory Effect of NO Production by LOBE

**Figure 3:**
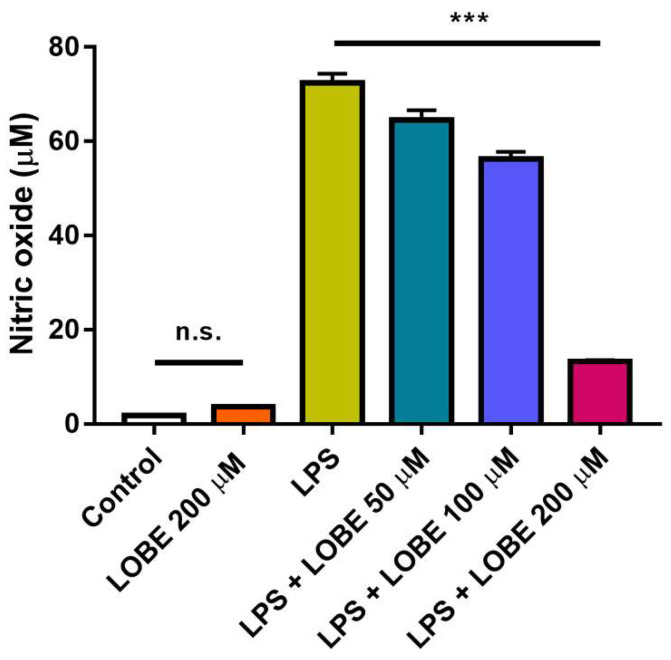
Inhibition of nitric oxide (NO) production by LOBE in lipopolysaccharide (LPS)-stimulated BMDMs. BMDMs were pre-treated with or without LOBE for 20 min, followed by stimulation or not with 0.1 µg/mL of LPS for 24 h. After 24 h, the NO-containing supernatant from each group was collected to quantify NO concentrations. Results are the mean ± SD of triplicate experiments: n.s., *** *p* < 0.001, significant difference as compared to the control group and each group by a one-way ANOVA followed by Tukey’s post hoc analysis.

To assess whether LOBE inhibit NO production, we quantified NO production in LPS-stimulated BMDMs and compared its changes after LOBE treatment. We first confirmed that LOBE alone did not induce any significant increases in NO production (Figure 3, control vs. LOBE 200 µM: 1.77 µM ± 0.13 vs. 3.60 µM ± 0.14, n.s.). In the LPS group, a significant increase in NO production was observed compared to the control group (Figure 3, control vs. LPS: 1.77 µM ± 0.13 vs. 72.36 µM ± 1.98, *** *p* < 0.001). This surge in NO was significantly decreased in LOBE treatment groups in a dose-dependent manner (Figure 3, LPS vs. LPS + LOBE 50 µM: 72.36 µM ± 1.98 vs. 64.60 µM ± 2.03, LPS + LOBE 50 µM vs. LPS + LOBE 100 µM: 64.60 µM ± 2.03 vs. 56.3 µM ± 1.47, LPS + LOBE 100 µM vs. LPS + LOBE 200 µM: 56.3 µM ± 1.47 vs. 13.23 µM ± 0.34, *** *p* < 0.001).

### 3.3. LOBE Decreased Pro-Inflammatory Gene Expressions in LPS-Induced BMDMs

**Figure 4:**
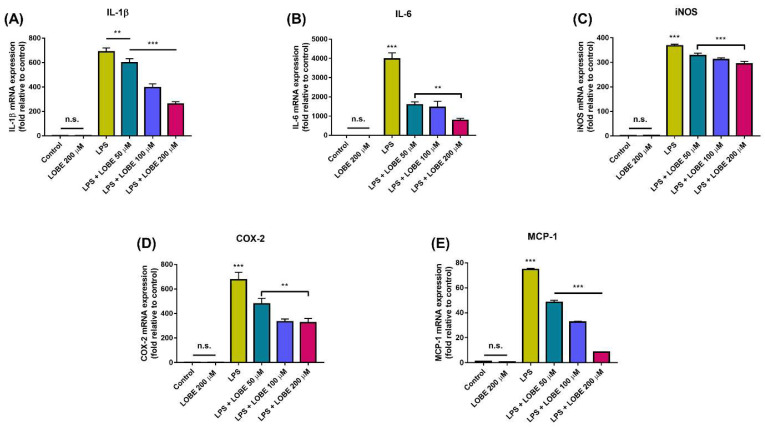
LOBE mediated down-regulation of gene expression of key pro-inflammatory molecules in LPS-induced BMDMs. The BMDMs were pre-treated with or without LOBE for 20 min, and then stimulated or not with 0.1 µg/mL of LPS for 24 h. The cells were then subjected to real-time quantitative polymerase chain reaction (RT-qPCR) analysis to detect mRNA expression levels of proinflammatory mediators (**A**) interleukin-1β (IL-1β) (**B**) IL-6, (**C**) inducible nitric oxide synthase (iNOS), (**D**) cyclooxygenase-2 (COX-2), and (**E**) monocyte chemoattractant protein-1 (MCP-1). The mRNA expressions of target genes were normalized to glyceraldehyde 3-phosphate dehydrogenase (GAPDH) mRNA. The expression level of control for all indicated genes was set at 1-fold, and relative fold change was calculated. Results are the mean ± SD of triplicate experiments: n.s., ** *p* < 0.01, *** *p* < 0.001, significant difference as compared to the control group and each group by a one-way ANOVA followed by Tukey’s post hoc analysis.

Next, we examined regulatory effect of LOBE on expression of pro-inflammatory cytokines and chemokines by measuring gene expression levels through RT-qPCR. LOBE itself did not induce pro-inflammatory gene expression (Figure 4, IL-1β, LOBE 200 µM: 1.24 ± 0.068; IL-6, LOBE 200 µM: 0.01 ± 0.070; iNOS, LOBE 200 µM: 2.12 ± 0.85; COX-2, LOBE 200 µM: 0.83 ± 0.17; MCP-1, LOBE 200 µM: 0.47 ± 0.02, n.s.). Remarkably, LPS-induced expression levels of pro-inflammatory genes IL-1β, IL-6, iNOS, COX-2, and MCP-1 were significantly reduced in all LOBE groups compared to that of LPS group, and the decrease was the most evident in 200 µM of LOBE (LPS + LOBE 200 µM: 260.04 ± 15.76, 780.43 ± 104.41, 293.94 ± 10.43, 325.19 ± 24.28, 8.38 ± 0.02, respectively, *** *p* < 0.001). Still, 50 µM of LOBE was sufficient to induce significant reduction in pro-inflammatory mRNA expression of IL-1β, IL-6, iNOS, COX-2, and MCP-1. (LPS + LOBE 50 µM 599.11 ± 16.90, ** *p* < 0.01, 1585.64 ± 159.39, 328.33 ± 8.73, 477.44 ± 47.10, 48.267 ± 1.69, *** *p* < 0.001, respectively).

### 3.4. LOBE Acts through MAPK Signaling Pathway by Decreasing Phosphorylation of ERK and JNK Signaling

**Figure 5:**
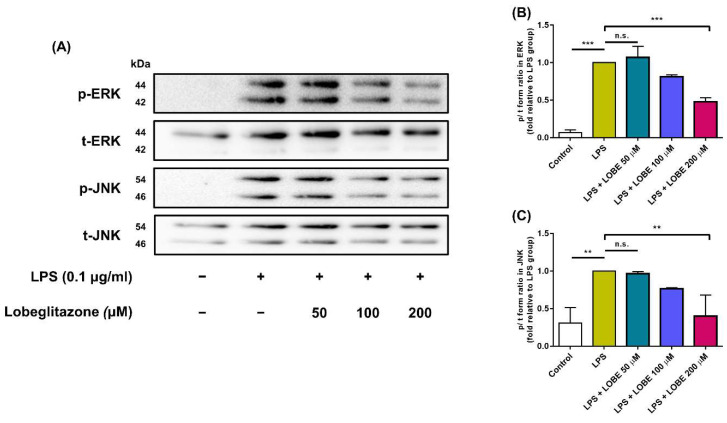
Effect of LOBE on phosphorylation level of extracellular regulatory kinase (ERK) (**A,B**) and c-Jun N terminal kinase (JNK) (A,**C**) of mitogen-activated protein kinase (MAPK) signaling pathway in LPS-stimulated BMDMs. BMDMs were pre-treated with or without LOBE for 20 min prior to 0.1 µg/mL of LPS treatment. After LPS stimulation, cells were harvested for Western blot analysis to detect changes in phosphorylation in ERK and JNK signaling. The ratio of band intensity of phosphorylated form over total form was quantified. The p/t volume for LPS group was set to 1-fold, and fold changes were calculated relative to LPS group. Results are the mean ± SD of triplicate experiments: n.s., ** *p* < 0.01, *** *p* < 0.001, significant difference as compared to the control group and each group by one-way ANOVA followed by Tukey’s post hoc analysis.

To test whether LOBE affect MAPK signaling pathway, phosphorylation state of ERK and JNK of MAPK signaling was quantified and compared. The phosphorylated form of ERK and JNK was significantly expressed in LPS group relative to that in control group (Figure 5A). In the LPS + 50 µM group, fold changes in phosphorylation state of ERK and JNK did not reach statistical significance when compared to the LPS group (Figure 5B–C, ERK, LPS vs. LPS + LOBE 50 µM: 1 ± 0 vs. 1.07 ± 0.033, JNK, LPS vs. LPS + LOBE 50 µM: 1 ± 0 vs. 0.96 ± 0.028, n.s.). However, a significant decrease in phosphorylation level of ERK and JNK was observed in the 200 µM LOBE group compared to that of the LPS group (ERK, LPS vs. LPS + LOBE 200 µM: 1 ± 0 vs. 0.48 ± 0.053, JNK, LPS vs. LPS + LOBE 200 µM: 1 ± 0 vs. 0.40 ± 0.028, ** *p* < 0.01).

## 4. Discussion

In this study, we tested anti-inflammatory effect of a TZD class drug, LOBE, in LPS-stimulated BMDMs. We demonstrate its effect on key pro-inflammatory mediators, and potential signaling pathways affected.

We first asked whether LOBE inhibit release of NO in activated macrophages. NO production is a hallmark of macrophage inflammation. Although NO is beneficial at moderate physiological concentration, sustained increased level of NO accumulates reactive oxygen species (ROS) and brings about upregulation of various pro-inflammatory gene expression in nearby cells. NO is produced by a number of distinct sets of NO synthases in macrophages, of which expression of iNOS is notable in the context of inflammation [2]. Aberrant iNOS and NO production are implicated in atherosclerosis and multiple sclerosis [28,29]. In our study, LOBE significantly inhibited LPS-induced NO production and expression of iNOS. This finding may suggest its potential antagonistic role of iNOS and NO secretion and therapeutic potential in diseases characterized by iNOS and NO production.

Since biological activities of NO include enhanced release of pro-inflammatory cytokines, we hypothesized LOBE can also regulate the expression of pro-inflammatory cytokines and chemokines. Our results show major pro-inflammatory effectors including IL-1β, IL-6, iNOS, COX-2, and MCP-1 were down-regulated by LOBE treatment. Previous research reported enhanced expression of IL-1 β and IL-6 in autoimmune and ischemic diseases [3,4]. Elevated COX-2 expression is implicated in inflammatory diseases, and its blockade is representative target of NSAID drugs [30,31]. Aberrant MCP-1 expression is a molecular marker of type 2 diabetes and other inflammatory diseases [6]. We present that LOBE inhibited pro-inflammatory gene expressions in macrophage, and this finding may propose potential repositioning of LOBE for inflammatory diseases.

To gain insight into the anti-inflammatory effect, we further examined the effect of LOBE on phosphorylation activities of two major MAPK proteins, ERK and JNK. Activation of MAPK pathways is involved in multiple cellular functions including inflammation by inducing transcription of multiple pro-inflammatory genes [32]. LOBE decreased ERK and JNK activation, and this result may explain the mechanism underlying suppression of pro-inflammatory gene expression.

A limitation in this study should be noted. Cytotoxicity in different organs needs to be demonstrated. Therefore, further in vivo study is needed to correlate with the proposed anti-inflammatory effect. Nonetheless, to the best of our knowledge, the present study firstly demonstrates the regulatory effect of a new TZD class, LOBE, on pro-inflammatory mediators in activated macrophages. We demonstrate that LOBE inhibited major cytokines and immune mediators through MAPK signaling pathway. The current study can propose the possibility of LOBE as a new anti-inflammatory agent in patients of inflammatory diseases.

## 5. Conclusions

LOBE inhibited NO production and major pro-inflammatory gene expressions in LPS-stimulated BMDMs. This anti-inflammatory effect is achieved possibly via inhibition of ERK and MAPK signaling pathway. Based on our results, we suggest potential application of LOBE in inflammatory diseases.

## Figures and Tables

**Table 1:** Nucleotide sequences of primers used in RT-qPCR.

Gene	Forward (5′ → 3′)	Reverse (5′ → 3′)
IL-1β	CCCTGCAGCTGGAGAGTGTGG	TGTGCTCTGCTTCAGAGGTGCT
IL-6	TTGTTGCTGTGGAGAAGCTGT	AACGTCACACACCAGCAGGTT
iNOS	CGGAGGAGAAGTGGGGTTTAGGAT	TGGGAGGCACTTGCGTTGATGG
COX-2	GACCAGATAAGGGCAAGCAC	CTTGTCTTTGACCCAGTAGC
MCP-1	ATGATTCTACCCACGGCAAG	CTGGAAGATGGTGATGGGTT
GAPDH	ATGATTCTACCCACGGCAAG	CTGGAAGATGGTGATGGGTT

## Data Availability

The data presented in this study are freely available on request from the corresponding author.
